# Supplementary material for: An updated evolutionary classification of CRISPR–Cas systems including rare variants
Source: Nat Microbiol. 2025 Nov 6;10(12):3346–61. doi: 10.1038/s41564-025-02180-8 (PMC12669027; doi:10.1038/s41564-025-02180-8)
Supplement: Supplementary file 1 — Supplementary Notes, Discussion and Tables 1–3. [file 41564_2025_2180_MOESM1_ESM.pdf]

nature microbiology

Supplementary information https://doi.org/10.1038/s41564-025-02180-8

An updated evolutionary classification of CRISPR–Cas systems including rare variants

In the format provided by the authors and unedited

Supplementary Notes

Modular organization of CRISPR-Cas systems

CRISPR-Cas systems show a characteristic modular organization that roughly corresponds to the 3 stages of adaptive immunity. The Cas proteins (a designation assigned to proteins performing central functions in CRISPR-Cas systems and/or represented in a broad variety of systems) together with ancillary proteins comprise four partially overlapping functional modules (Fig. 1) [1]. The core of the adaptation module consists of the integrase Cas1, the key enzyme involved in spacer insertion, and Cas2, which forms the structural scaffold of the adaptation complex. In addition, the adaptation modules of many type I CRISPR-Cas systems include a Cas4 nuclease, and that of subtype II-A includes Csn2, a structural subunit of the adaptation complex [2-5](Extended Data Fig. 1-4). The adaptation modules of many type III systems encompass a reverse transcriptase (RT) that is responsible for spacer insertion via reverse transcription of RNA targets [6,7].

The expression-processing module in most class 1 systems consists of a single protein, Cas6, that catalyzes the processing of pre-crRNA into mature crRNAs [8]. Class 2 systems show a greater diversity of processing mechanisms. In type II and some type V systems, processing is mediated by a trans-activating crRNA (tracrRNA) along with the bacterial RNase III (experimentally demonstrated for type II and predicted for type V), which is not encoded in CRISPR-*cas* loci and has other functions in bacterial cells [9,10]. In most type V systems and apparently in all type VI systems, crRNA maturation is catalyzed via catalytic sites of the Cas12 and Cas13 effector proteins distinct from the sites involved in the target cleavage [11,12].

The effector (interference) module is responsible for the crRNA-dependent recognition and (typically) cleavage of the target DNA or RNA. Class 1 effector modules encompass elaborate supramolecular complexes (known as CRISPR-Associated Complex for Antiviral Defense, Cascade, in the case of type I) consisting of different combinations of Cas5, Cas7, Cas8 (type I), Cas10 (type III) and Cas11 subunits (Fig. 1) [1]. In type I systems, the Cascade complex, once bound to the target DNA, recruits the helicase-nuclease Cas3, which unwinds and shreds the target [13]. In type III systems, the HD nuclease domain of Cas10 cleaves the target DNA whereas Cas7 cleaves the target RNA [14]. Most of the Cas10 proteins in type III CRISPR-Cas systems are active polymerases-cyclases that synthesize a signaling nucleotide derivative, which binds to the sensor domain (typically, CARF, CRISPR-associated Rossmann fold) of an ancillary protein that also contains an effector, most often a nuclease, that induces dormancy or death of the infected cell [14]. Most type IV systems lack identifiable nuclease domains in effector proteins, but those of subtype IV-A instead encompass the CasDinG helicase (a distinct branch in the DinG family) that is involved in transcription repression [15], whereas subtype IV-B systems typically include CysH, phosphoadenosine 5'-phosphosulfate reductase, the role of which remains enigmatic. Many type IV systems, especially within subtype IV-B`, lack associated CRISPR arrays. The class 2 effector modules consist of a single, multidomain protein, Cas9 (type II), Cas12 (type V) or Cas13 (type VI), that is fully responsible for the target recognition and cleavage [1,16] which makes class 2 Cas nucleases tools of choice for genome editing. The ancillary module of CRISPR-Cas systems is a collection of CRISPR-linked proteins found in smaller subsets of CRISPR-Cas systems, primarily of type III, including the enzymes involved in the built in (oligo)nucleotide signaling pathway (Fig. 1 and see below).

Supplementary Discussion

The origins of both classes of CRISPR-Cas systems appear to follow the complexification scenario (Extended Data Fig. 10). The most plausible route to class 1 CRISPR-Cas systems leads from defense systems that encompass a 'minimal' Cas10 homolog consisting mostly of the polymerase/cyclase domain (denoted mCPol, after Minimal CRISPR Polymerase) and a CARF-HEPN protein resembling type III CRISPR components that mediate promiscuous RNA cleavage and cell dormancy or death in response to cOA [1,2]. Recently, an mCPol variant combined with a distinct effector has been shown to synthesize a second messenger, 2',3'-c-di-AMP [3]. Thus, although not yet studied experimentally, these putative ancestors of class 1 CRISPR-Cas effector complexes can be confidently predicted to function via the same mechanism as the CBASS (Cyclic oligonucleotide-based antiphage signaling system) defense systems [4] where the effector HEPN nuclease is activated by a cyclic nucleotide second messenger synthesized by the cyclase in response to infection.   However, the CBASS cyclase is unrelated to mCPol and Cas10. Under this scenario, perhaps counterintuitively, the complex type III CRISPR-Cas systems that combine direct inactivation of target genomes by cleavage with fine-tuned dormancy induction or programmed cell death (PCD) executed via the built-in cOA signaling pathway were the first to evolve in class 1. The path from the ancestral, CBASS-like systems to type III CRISPR-Cas would involve substantial complexification including proliferation of RNA-binding domains (Cas5, Cas6, Cas7) that have the same RRM (RNA Recognition Motif) fold as the catalytic domain of Cas10. The genes encoding these RRM domain proteins might have evolved by a series of duplications, with subsequent extensive divergence, capture of additional domains, including HD nuclease, and diversification of sensors and effectors [2,5,6].

In contrast, under this scenario, subsequent evolution of class 1 CRISPR-Cas systems seems to have involved primarily reduction and streamlining (Extended Data Fig. 8). In particular, type I, by far the most common type of CRISPR-Cas in both bacteria and archaea, most likely, evolved from type III. Subtype I-D, which encodes an inactivated Cas10 and no signaling pathway components, represents a likely intermediate on this evolutionary path [2,7]. The key events in type I CRISPR-Cas evolution apparently were the loss of the signaling pathway and the capture of the effector helicase-nuclease Cas3. Thus, type I systems appear to have mostly lost the capacity of type III systems to induce dormancy or PCD, while retaining and enhancing the capacity to destroy target genomes. In type III systems, the activity of the ancestral CBASS-like modules that apparently targeted and cleaved RNA via the HEPN domain was augmented by adding the DNA cleavage capacity through the capture of the HD nuclease domain. In type I systems, the efficiency of DNA-targeting was further enhanced through the acquisition of the Cas3 helicase-nuclease whereas RNA cleavage resulting in toxicity was lost. Although eliminating the flexibility of immune response, the switch from growth arrest invader clearance might have reduced the fitness cost of type I systems, underpinning, in part, their evolutionary success.

Evolution of subtype III-E from the III-D2 variant involved a distinct route of streamlining, namely, the loss of Cas10 and Cas6, accompanied by fusion of three Cas7 paralogs in III-D2, with a fourth Cas7 and Cas11 additionally fused in III-E [2,8]. As in other streamlined CRISPR-Cas systems, reductive evolution in subtype III-E was complemented by acquisition of accessory proteins which in this case perform regulatory functions [9-11].

More radical reductive evolution apparently led from type III to type IV [12,13]. Subtype IV-A systems, the most common within type IV, as well as subtype IV-B, lack effector nucleases and the counterpart of

Cas11, and encompass a large effector complex subunit that is much smaller than Cas10. Subtype IV-C systems contain a larger Cas10 counterpart that retains the HD nuclease domain, suggesting that these are intermediates in the evolution of type IV from type III [13].

An even more dramatic reduction of type III CRISPR-Cas systems apparently produced miniature CRISPR derivatives, HRAMP (found in Halobacteria) [14] and ARAMP (in Asgard archaea) [15]. The HRAMP and ARAMP loci are associated with neither adaptation modules nor CRISPR arrays. It currently remains unclear whether these CRISPR-derived modules interact with crRNA or not, but in case they do, they differ from type III systems to such an extent as to qualify as CRISPR-Cas types VIII and IX.

Exaptation of CRISPR-Cas systems by MGEs also was accompanied by loss of components [13], and in a notable parallel, type IV systems that evolved via the reductive route are found primarily in plasmids and phages, and apparently have been exapted for competition between these MGEs.

Compared to class 1, class 2 CRISPR-Cas systems have a simpler organization, and their origin and evolution are better understood [2]. As pointed out above, type II and type V CRISPR-Cas effectors evolved from RNA-guided nucleases encoded by transposons of the IS*200*/IS*605* family, IscB and TnpB, respectively [16,17 Altae-Tran, 2023 #1887]. However, the inferred evolutionary histories of these two CRISPR-Cas types differ substantially because type II appears to be monophyletic, with Cas9 evolving from IscB only once, whereas Cas12s of different type V subtypes evolved from TnpB derivatives on numerous, independent occasions [16,18,19]. Notably, subtype V-K and V-F CRISPR-Cas systems with inactivated Cas12 nucleases were recruited as CASTs, the former by a group of Tn7-like transposons [16] and the latter by Mu-like transposons [18]. Many other type V variants are encoded by plasmids and viruses where their roles remain to be elucidated [18].

The type VI effector, Cas13, that contains a tandem of HEPN RNase domains has long been thought to have evolved from a HEPN-containing toxin component of a toxin-antitoxin (TA) module [20]. Recently, this connection was explored in greater detail and clarified [21]. Among the identified TA modules, AbiF, a type III TA [22], consisting of a HEPN toxin and an RNA antitoxin, turned out to be most closely related to Cas13 and is the likely ancestor of Cas13. Evolution of Cas13 from AbiF involved duplication of the HEPN domain that might have occurred twice independently, in the ancestor of Cas13b and in the common ancestor of the rest of the type VI effectors, and capture of additional domains interacting with the target RNA.

The common trend in the evolution of all class 2 effectors from their nuclease ancestors involved substantial increase in the size and complexity of the effector proteins, with the new domains providing for complex formation with target DNA or RNA [21,23]. Thus, in the evolution of class 2 systems, complexification seems to be the predominant trend whereas reductive evolution is less common than it is in class 1. A prominent manifestation of complexification in CRISPR-Cas evolution is the acquisition of ancillary genes encoding accessory or regulatory proteins found to be associated with many CRISPR-Cas systems, most notably, those of type III, but also with type II and type VI systems.

CRISPR-Cas adaptation modules, originally apparently derived from a casposon, a self-replicating transposon with a transposase homologous to Cas1[24], are extensively recombined with different effector modules. It has been demonstrated on multiple occasions that effector modules could be exchanged *in situ*, while the adaptation module and the CRISPR array remained in the same place [25]. Most of these

cases were described for class 1 systems, but apparently, similar recombination of modules occurs in class 2 loci as well (Extended Data Fig. 9A). By tallying the co-occurrence of *cas1* with distinct effector genes (*cas8*, *cas9*, *cas10* or *cas12*) within the same locus, we detected 1-2 between-modules recombination events per 100 distinct adaptation modules (See Methods and Extended Data Fig. 9B). This shuffling of the CRISPR-Cas modules, recently described nuclease exchanges [18] and the extreme divergence of Cas8 subunits of type I effector complexes, as demonstrated by comparison of structural models and sequence profiles of these protein families (Extended Data Fig. 10), might be driven by selection for Acr escape (Fig. 6).

Reports of CRISPR-Cas systems recruitment for regulation of gene expression are growing [26]. There seem to be two distinct routes of evolution towards regulatory functions. The first route involves acquisition of additional crRNA-like regulatory elements by CRISPR-*cas* loci. Examples include the long form of tracrRNA in II-A systems that redirects Cas9 to regulate its own promoter [27], regulation of type VIII TA modules (CreTA) by I-B Cascade in Halobacteria resulting in safeguarding of the CRISPR-*cas* locus [28], and regulation of developmental genes in *Myxococcus* by I-B Cascade as well [29]. The discovery of stand-alone crRNA-like elements in diverse CRISPR-*cas* loci implies that this type of regulation is much more common than previously suspected [30]. The second route of regulatory function evolution is reductive, involving loss of nuclease activity and, often, the CRISPR array. Several such instances have been described for derivatives of type V-F systems [18]. Furthermore, most type IV as well as V-M systems likely down-regulate plasmid gene expression as the main mechanism of inter-plasmid competition [13]. Finally, it has been predicted by genome analysis [30] and subsequently demonstrated experimentally [31] that some phages employ CRISPR mini-arrays to suppress expression of host *cas* genes.

Supplementary Tables

Supplementary Table 1. Targeting diversity of CRISPR-Cas systems

**Type**	**Subtype or variant**	**Active nuclease**	**Sequence motif***
**DNA targeting**			
I	A1, B1, C1, D1, F1, F2, E1, G	Cas3 (HD domain)	H..HD..D
V	A-F, H-J, L, N, Q	Cas12 (RuvC domain)	D..E..D
II	A-D	Cas9 (RuvC domain)	D..E..D
I	I-E3	HNH nuclease (fused to Cas5)	H..N..H
I	I-E4	PD-DExK nuclease (fused to Cas11)	D..ExK
I	I-F4	HNH nuclease (fused to Cas8)	H..N..H

III	A, B, D1 (some), C, F, G	Cas10 (HD domain)	HD..D..H
IV	C	Cas8-IVc (HD domain)	HD..D..H
IV	A2	DinG (HNH nuclease domain)	H..N..H
**RNA targeting**			
III	A, B, D	Cas7	Varied
III	E	Cas7-11	Several aspartates
V	G	Cas12g (RuvC domain)	D..E..D
VI	A, B1, B2, C, D, E, F	Two HEPN domains	RxxxxH
VII	A	Cas14 (β-CASP domain)	HxHxxH..D..H..H
**Collateral activity**			
I	E2		See above
V	A1, A2		See above
VI	A, B1, B2, C, D		See above
**Inactivated**			
IV	A1, B		
I	B2, C2, D2, F3		
V	M, K, O, P, B3		

* In the motif signature "." means any number of any amino acids between conserved residues and "x" means any amino acids for fixed motif length

Supplementary Table 2. Protein components of the built-in signaling pathways of type III CRISPR-Cas systems

New name*	Old names	Superfamily	References	Functions #	Comment
**cOA pathway components**					
Crf1	Csm6	CARF+HEPN	[1-4]	s,e,r	Typically, binds and cleaves cA6, but also cA5 and cA4; HEPN effector domain is an RNase that is activated by cOA binding; CARF domain cleaves cA6, but also cA5 and cA4. Mostly associated with type III-A systems
Crf2	Csx1; CARF1	CARF+HEPN	[4-7]	s,e	Binds cA4; HEPN domain is an RNase induced by cA4 binding; in some proteins. HEPN domain also has ring nuclease activity
Crf4	Can1;Can2; Card1; CARF4	CARF+PD-(D/E)xK	[7,8]		Binds cA4; PD-(D/E)xK domains are nucleases that are activated by cA4 binding and degrade DNA an/or RNA
Crf5	CARF5, CARF_m13, Cad1	CARF or CARF+ADA	[9,10]	s,e,r	Binds cA4 or cA6; some are fused to adenosine deaminase (ADA) domain which, upon activation, converts ATP to ITP.
Crf6	CARF_m1	CARF+PIN	[9]	s,e,r	PIN domain is a RNase; typically, not associated with CRISPR-systems.
Crf7	Crn1, CARF7, Cami1	CARF + RelE	[11]	s,e,r	Cami1 binds cA4 activating RelE RNases which degrades mRNA in the ribosome. Many other members of the family likely have ring nuclease activity. Crn1 cleaves cA4; Often fused to effector domains of RelE (RNase) and DUF2103 families.
Crf8	Csm6-2; CARF_m11	CARF+HEPN	[12]	s,e	Binds cA6; HEPN domain is an RNase that is activated by cA6 binding.
Crf9	CARF9, Csx1	CARF+HEPN	[9]	s,e,r	Some have ring nuclease activity.
Crf10	CARF_m3	CARF+PIN	[9]	s,e	Archaea-specific; PIN is a predicted RNase
Crf11	CARF_m3	CARF+PIN	[9]	s,e	Archaea-specific; PIN is a predicted RNase
CalpL		SAVED+Lon	[9,13,14]	s,e,r	Binds cA4 activating the Lon-like protease domain which cleaves anti-sigma factor CalpT triggering anti-

					viral transcriptional response; SAVED domain is also a ring nuclease
NucC	NucC	PD-(D/E)xK	[15,16]	s,e	cA3 is bound by a subdomain of NucC and activates the dsDNA nuclease
Cam1	Cam1; CARF8	CARF+trans membrane domain	[10]	s,e	Binds cA4; upon activation, causes membrane depolarization
Cam2	Cam2; Mem_04	Receiver domain	[12,17]	s,e	Predicted to bind cA3; membrane protein
Cam3	Cam3; Mem_01	-	[12,17]	s,e	Membrane protein; function unclear
Csx21	Csx21	Csx21	[12]	s,e	Membrane protein; function unclear
Csx18	Csx18	Csx18	[12]	s,e	Membrane protein; function unclear
Csx23	Csx23	Csx23	[15]	s,e	Binds cA4, fused to an N-terminal transmembrane domain
Crn2	Crn2; AcrIII	DUF1874	[18]	r	Anti-CRISPR protein, often encoded in viruses, inhibits type III CRISPR-Cas systems
Crn3	Csx3	Csx3	[19]	r	Degrades cA4
Crn4	Unk_01		[19]	r	Degrades cA3, cA4, cA6
Crn5	-	SAVED	[20]	r	Degrades cA4
Csx15	Csx15	Csx15	[9,21]	r	Binds and in some cases degrades cA4
Csx16	Csx16	Csx16	[9] [21]	r	Degrades cA4
Csx20	Csx20	Csx20	[9] [21]	r	Degrades cA4
**SAM-AMP pathway components**					
CorA	CorA	CorA	[22]	s,e	Membrane protein homologous to the magnesium channel CorA; Activated by the SAM-AMP and causes membrane depolarization
Csd1	-	SAM lyase	[22]	d	Cleaves SAM-AMP
Csd2	-	DHH	[22]	d	Cleaves SAM-AMP

Csd3	-	DEDD	[22]	d	Predicted to cleave SAM-AMP

Asterisks denote new names for CARF superfamily (Crf) which are based on the clustering dendrogram (Extended Data Figure7), which covered only the major clades in the phylogeny of the CARF domains [23](Data file 4). Pound sign denotes the function identified or predicted for the family members: s, sensor, e, effector, r, ring nuclease, d, SAM-AMP degradation. Crf3 is not included because the CARF domain-containing proteins in this clade are typically not associated with CRISPR-Cas systems [9]. New names can be used along with legacy names. "Csx" are legacy names which might be later replaced by other gene names based on experimental characterization of respective proteins, eg. to Crn for ring nucleases. Csd, designation proposed here for CRISPR SAM-AMP-degrading. Abbreviations (other than those explained in the text and comments): Csd, designation proposed here for CRISPR SAM-AMP-degrading; SAVED – "SMODS-associated and fused to various effector domain" is distantly related to CARF; DHH – nuclease of DHH family, also known as NrN nuclease; DEDD – DnaQ-like nuclease of RNase H superfamily.

Supplementary Table 3. Updates of CRISPR system classification nomenclature conflicting with previously published subtypes and variants

Previous publication	Subtypes or variants	Current classification	Comment
[24]	II-C2	II-D	Based on phylogenetic analysis provided in this work
[25]	Subtype IV-E<br>Variants IV-A1, IV-A2, IV-A3	All remain in IV-A	Based on analysis in [26]
[25]	Subtype IV-D	Unclassified	RecD associated subtype of hybrid origin. The most conserved Cas7 component is from IV-B clade. Based on analysis in [26];
[27]	VI-E, VI-F, VI-G, VI-H, VI-I	All assigned to in IV-B	Based on sequence comparisons provided in this work
[28]	I-F1	IF-4	I-F1 was assigned previously to a different system [24]

References

1 Jia, N., Jones, R., Yang, G., Ouerfelli, O. & Patel, D. J. CRISPR-Cas III-A Csm6 CARF Domain Is a Ring Nuclease Triggering Stepwise cA(4) Cleavage with ApA>p Formation Terminating RNase Activity. Mol Cell 75, 944-956 e946, doi:10.1016/j.molcel.2019.06.014 (2019).

2 Smalakyte, D. et al. Type III-A CRISPR-associated protein Csm6 degrades cyclic hexa-adenylate activator using both CARF and HEPN domains. Nucleic Acids Res 48, 9204-9217, doi:10.1093/nar/gkaa634 (2020).

3 Garcia-Doval, C. et al. Activation and self-inactivation mechanisms of the cyclic oligoadenylate-dependent CRISPR ribonuclease Csm6. Nat Commun 11, 1596, doi:10.1038/s41467-020-15334-5 (2020).

4 Ding, J., Schuergers, N., Baehre, H. & Wilde, A. Enzymatic properties of CARF-domain proteins in Synechocystis sp. PCC 6803. Front Microbiol 13, 1046388, doi:10.3389/fmicb.2022.1046388 (2022).

5 Han, W., Pan, S., Lopez-Mendez, B., Montoya, G. & She, Q. Allosteric regulation of Csx1, a type IIIB-associated CARF domain ribonuclease by RNAs carrying a tetraadenylate tail. Nucleic Acids Res 45, 10740-10750, doi:10.1093/nar/gkx726 (2017).

6 Molina, R. et al. Structure of Csx1-cOA(4) complex reveals the basis of RNA decay in Type III-B CRISPR-Cas. Nat Commun 10, 4302, doi:10.1038/s41467-019-12244-z (2019).

7 McMahon, S. A. et al. Structure and mechanism of a Type III CRISPR defence DNA nuclease activated by cyclic oligoadenylate. Nat Commun 11, 500, doi:10.1038/s41467-019-14222-x (2020).

8 Rostol, J. T. et al. The Card1 nuclease provides defence during type III CRISPR immunity. Nature 590, 624-629, doi:10.1038/s41586-021-03206-x (2021).

9 Makarova, K. S. et al. Evolutionary and functional classification of the CARF domain superfamily, key sensors in prokaryotic antivirus defense. Nucleic Acids Res 48, 8828-8847, doi:10.1093/nar/gkaa635 (2020).

10 Baca, C. F. et al. The CRISPR effector Cam1 mediates membrane depolarization for phage defence. Nature 625, 797-804, doi:10.1038/s41586-023-06902-y (2024).

11 Mogila, I. et al. Ribosomal stalk-captured CARF-RelE ribonuclease inhibits translation following CRISPR signaling. Science 382, 1036-1041, doi:10.1126/science.adj2107 (2023).

12 Hoikkala, V., Graham, S. & White, M. F. Bioinformatic analysis of type III CRISPR systems reveals key properties and new effector families. Nucleic Acids Res 52, 7129-7141, doi:10.1093/nar/gkae462 (2024).

13 Binder, S. C. et al. The SAVED domain of the type III CRISPR protease CalpL is a ring nuclease. Nucleic Acids Res 52, 10520-10532, doi:10.1093/nar/gkae676 (2024).

14 Smalakyte, D., Ruksenaite, A., Sasnauskas, G., Tamulaitiene, G. & Tamulaitis, G. Filament formation activates protease and ring nuclease activities of CRISPR Lon-SAVED. Mol Cell 84, 4239-4255 e4238, doi:10.1016/j.molcel.2024.09.002 (2024).

15 Gruschow, S., Adamson, C. S. & White, M. F. Specificity and sensitivity of an RNA targeting type III CRISPR complex coupled with a NucC endonuclease effector. Nucleic Acids Res 49, 13122-13134, doi:10.1093/nar/gkab1190 (2021).

16 Lau, R. K. et al. Structure and Mechanism of a Cyclic Trinucleotide-Activated Bacterial Endonuclease Mediating Bacteriophage Immunity. Mol Cell 77, 723-733 e726, doi:10.1016/j.molcel.2019.12.010 (2020).

17 Shmakov, S. A., Makarova, K. S., Wolf, Y. I., Severinov, K. V. & Koonin, E. V. Systematic prediction of genes functionally linked to CRISPR-Cas systems by gene neighborhood analysis. Proc Natl Acad Sci U S A 115, E5307-E5316, doi:10.1073/pnas.1803440115

1803440115 [pii] (2018).

18 Samolygo, A., Athukoralage, J. S., Graham, S. & White, M. F. Fuse to defuse: a self-limiting ribonuclease-ring nuclease fusion for type III CRISPR defence. Nucleic Acids Res 48, 6149-6156, doi:10.1093/nar/gkaa298 (2020).

19 Athukoralage, J. S. et al. Tetramerisation of the CRISPR ring nuclease Crn3/Csx3 facilitates cyclic oligoadenylate cleavage. Elife 9, doi:10.7554/eLife.57627 (2020).

20 Orzechowski, M. H., V., Chi, H., McMahon, S., Gloster, T. & White, M. F. A viral SAVED protein with ring nuclease activity degrades the CRISPR second messenger cA4. https://www.biorxiv.org/content/10.1101/2025.07.01.662501v1 (2025).

21 Hoikkala, V., Chi, H., Grüschow, S., Graham, S. & White, M. F. Diversity and abundance of ring nucleases in type III CRISPR-Cas loci. https://www.biorxiv.org/content/10.1101/2024.09.24.614671v1 (2024).

22 Chi, H. et al. Antiviral type III CRISPR signalling via conjugation of ATP and SAM. Nature 622, 826-833, doi:10.1038/s41586-023-06620-5 (2023).

23 Makarova, K. S. et al. An updated evolutionary classification of CRISPR-Cas systems including rare variants. https://doi.org/10.5281/zenodo.15882620 (2025).

24 Makarova, K. S. et al. Evolutionary classification of CRISPR-Cas systems: a burst of class 2 and derived variants. Nat Rev Microbiol 18, 67-83, doi:10.1038/s41579-019-0299-x (2020).

25 Pinilla-Redondo, R. et al. Type IV CRISPR-Cas systems are highly diverse and involved in competition between plasmids. Nucleic Acids Res 48, 2000-2012, doi:10.1093/nar/gkz1197 (2020).

26 Moya-Beltrán, A. et al. Evolution of Type IV CRISPR-Cas systems: insights from CRISPR loci in Integrative Conjugative Elements of Acidithiobacillia CRISPR J (2021).

27 Hu, Y. et al. Metagenomic discovery of novel CRISPR-Cas13 systems. Cell Discov 8, 107, doi:10.1038/s41421-022-00464-5 (2022).

28 Hirano, S., Altae-Tran, H., Kannan, S., Macrae, R. K. & Zhang, F. Structural determinants of DNA cleavage by a CRISPR HNH-Cascade system. Mol Cell 84, 3154-3162 e3155, doi:10.1016/j.molcel.2024.07.026 (2024).
